# Immunohistochemistry study of tumor vascular normalization and anti-angiogenic effects of sunitinib versus bevacizumab prior to dose-dense doxorubicin/cyclophosphamide chemotherapy in HER2-negative breast cancer

**DOI:** 10.1007/s10549-021-06470-7

**Published:** 2021-12-20

**Authors:** Kritika Yadav, Joline Lim, Joan Choo, Samuel Guan Wei Ow, Andrea Wong, Matilda Lee, Ching Wan Chan, Mikael Hartman, Siew Eng Lim, Natalie Ngoi, Siau Wei Tang, Yvonne Ang, Gloria Chan, Wan Qin Chong, Hon Lyn Tan, Sing Huang Tan, Boon Cher Goh, Soo Chin Lee

**Affiliations:** 1 Present Address: Department of Pathology, Dr. D Y Patil Medical College, Navi Mumbai, India; 2 Cancer Science Institute, National University of Singapore, Singapore, Singapore; 3 Department of Haematology-Oncology, National University Cancer Institute, National University Health System, Singapore, Singapore; 4 Department of Surgery, National University Cancer Institute, National University Health System, Singapore, Singapore

**Keywords:** Anti-angiogenic, Bevacizumab, HER2-negative breast cancer, Sunitinib, Vascular normalization

## Abstract

**Purpose:**

Tumor angiogenesis controlled predominantly by vascular endothelial growth factor and its receptor (VEGF-VEGFR) interaction plays a key role in the growth and propagation of cancer cells. However, the newly formed network of blood vessels is disorganized and leaky. Pre-treatment with anti-angiogenic agents can “normalize” the tumor vasculature allowing effective intra-tumoral delivery of standard chemotherapy. Immunohistochemistry (IHC) analysis was applied to investigate and compare the vascular normalization and anti-angiogenic effects of two commonly used anti-angiogenic agents, Sunitinib and Bevacizumab, administered prior to chemotherapy in HER2-negative breast cancer patients.

**Methods:**

This prospective clinical trial enrolled 38 patients into a sunitinib cohort and 24 into a bevacizumab cohort. All received 4 cycles of doxorubicin/cyclophosphamide chemotherapy and pre-treatment with either sunitinib or bevacizumab. Tumor biopsies were obtained at baseline, after cycle 1 (C1) and cycle 4 (C4) of chemotherapy. IHC was performed to assess the tumor vascular normalization index (VNI), lymphatic vessel density (LVD), Ki67 proliferation index and expression of tumor VEGFR2.

**Results:**

In comparison to Bevacizumab, Sunitinib led to a significant increase in VNI post-C1 and C4 (*p* < 0.001 and 0.001) along with decrease in LVD post-C1 (*p* = 0.017). Both drugs when combined with chemotherapy resulted in significant decline in tumor proliferation after C1 and C4 (baseline vs post-C4 Ki67 index *p* = 0.006 for Sunitinib vs *p* = 0.021 for Bevacizumab). Bevacizumab resulted in a significant decrease in VEGFR2 expression post-C1 (*p* = 0.004).

**Conclusion:**

Sunitinib, in comparison to Bevacizumab showed a greater effect on tumor vessel modulation and lymphangiogenesis suggesting that its administration prior to chemotherapy might result in improved drug delivery.

**Trial registry:**

ClinicalTrials.gov: NCT02790580 (first posted June 6, 2016).

## Introduction

As per global cancer statistics 2018, breast cancer is the most commonly diagnosed cancer in females, with an incidence rate of 46.2 per 100,000 and representing 24.2% of the total female cancer burden globally. It is also the leading cause of cancer death with a mortality rate of 15% [1]. Multiple studies have highlighted the key role of tumor vascularization in facilitating tumor growth, progression and metastasis of various solid tumors including breast cancer [2, 3]. Bevacizumab is an anti-vascular endothelial growth factor (VEGF) monoclonal antibody, while Sunitinib is an orally administered small molecule receptor tyrosine kinase inhibitor that exerts its action by targeting the vascular endothelial growth factor receptors (VEGFR). Both agents have been studied in clinical trials in combination with chemotherapy in breast cancer. Disappointingly, although both anti-angiogenic agents have shown promising preclinical results, their effects in breast cancer when combined with chemotherapy have been conflicting in the clinic. This could be in part attributed to the fact that optimal dosing schedule of these drugs in combination with chemotherapy are yet to be determined [4–6].

A major pathway involved in angiogenesis is the release of VEGF from hypoxic tumor cells and its binding to the VEGFR expressed on the vascular and lymphatic endothelial cells, leading to endothelial cell proliferation and migration. However, the newly formed blood vessels in a growing tumor are dilated, leaky and poorly organized with no pericyte covering. These “immature” vessels have variable blood flow resulting in sub-optimal delivery of chemotherapeutic drugs. The careful and judicious use of anti-angiogenic drugs can “normalize” these abnormally structured blood vessels within the tumor leading to more efficient drug delivery [7]. In addition, VEGFR2 is expressed on various tumors including breast cancer and is responsible for the autocrine and paracrine effect of VEGF resulting in tumor cell survival and proliferation [8].

We hypothesize that pre-treatment rather than concurrent treatment with sunitinib or bevacizumab prior to standard chemotherapy in human epidermal growth factor receptor 2 (HER2)-negative breast cancer, along with a lower dose of the anti-angiogenic agent, will improve and “normalize” the tumor vasculature making it more efficient for intra-tumoral chemotherapy drug delivery. We enrolled patients into a prospective clinical trial and obtained serial tumor biopsies at baseline, during and after chemotherapy to assess and compare the vascular normalization and anti-angiogenic effects of sunitinib versus bevacizumab. Vascular normalization, lymphatic density, tumor proliferation index and activated VEGFR2 status of tumor cells were studied in the tumor specimens.

## Patients and methods

### Study population

Patients were enrolled into a prospective, phase II open label, single-arm study conducted at the National University Cancer Institute, Singapore (NCIS). Eligibility criteria included female patients aged ≥ 18 years with newly diagnosed and histologically confirmed HER2-negative breast cancer. HER2 negativity was defined as HER2 score 0 or 1 + on immunohistochemistry (IHC) or HER2 IHC 2 + but HER2 Fluorescence In Situ Hybridization (FISH)-negative (HER2/CEP17 ratio < 2.0 with gene copy number < 4.0 signals/ cell) [9]. Other inclusion criteria were measurable primary tumor ≥ 2 cm, Eastern Cooperative Oncology Group (*ECOG*) performance 0 or 1, absolute neutrophilic count ≥ 1.5 × 10^9^/L, platelets ≥ 100 × 10^9^/L, serum total bilirubin ≤ 1.5 × upper limit of normal (ULN), alanine and aspartate aminotransferase ≤ 2.5 × ULN, serum creatinine ≤ 1.5 × ULN and left ventricular ejection fraction ≥ 50%. Patients with clinically detectable second primary malignancy, symptomatic brain metastasis, and known history of systemic connective tissue diseases were excluded from the study. Signed written informed consent was taken from all patients before enrollment. The clinical trial was conducted in accordance with local regulatory requirements and approved by the institutional ethics review board.

### Treatment plan and study design

The study aimed to determine the effect of pre-treatment with low-dose anti-angiogenic agent prior to chemotherapy as a strategy to normalize tumor vasculature. Patients were enrolled into two sequential Cohorts to study two different classes of anti-angiogenic agents: Sunitinib Cohort to evaluate a small molecule tyrosine kinase inhibitor against VEGFR, and Bevacizumab Cohort to evaluate a monoclonal antibody against VEGF. All subjects received 4 cycles of dose-dense doxorubicin/cyclophosphamide (ddAC) chemotherapy every 2 weeks (doxorubicin 60 mg/m^2^ and cyclophosphamide 600 mg/m^2^) supported by prophylactic pegfilgrastim 6 mg administered subcutaneously 24–48 h after each cycle of chemotherapy. Patients enrolled into the Sunitinib Cohort were pre-treated with oral sunitinib 12.5 mg daily for 7 days prior to cycle 1 ddAC and for 5 days prior to cycles 2, 3 and 4 ddAC. Patients enrolled into the Bevacizumab Cohort received intravenous bevacizumab 5 mg/kg, 7 days prior to each chemotherapy cycle. Dosing of sunitinib was determined through a phase Ib/II study by Wong et al. and dosing of bevacizumab was determined based on extrapolation from a preclinical study by Gaustad et al. [10, 11]. Primary endpoint of the study was rate of pathological complete response (pCR) post neoadjuvant chemotherapy, defined as absence of invasive cancer in both breast and axillary lymph nodes time of surgery, with pharmacodynamic biomarker exploration through IHC as a secondary endpoint.

For each patient, detailed history was recorded. Physical examination, radiological staging with computed tomography (CT) scan or CT-PET scan and laboratory evaluation was done prior to initiating treatment. Patients were evaluated before each new cycle of chemotherapy to monitor adverse effects from treatment and to measure tumor response. Response to treatment was assessed clinically according to the Response Evaluation Criteria in Solid Tumors (RECIST) version 1.1 criteria [12].

### Tumor core biopsies for immunohistochemistry (IHC) studies

Tumor core biopsies were obtained from the primary breast tumor under ultrasound guidance at baseline, 2 weeks after cycle 1 ddAC but before cycle 2 ddAC (post-C1) and about 2 weeks after completion of 4 cycles of ddAC (post-C4). Tumor cores were fixed in formalin for further histological and IHC analysis.

### Immunohistochemistry studies

Tumor biopsies taken at each time point were processed into paraffin blocks. Hematoxylin and Eosin (H&E) staining was done to identify and assess the tumor content. Biopsies which showed tumor content ≤ 10% were excluded from further evaluation and staining. IHC was performed on consecutive slides using the Leica Bond Max automated platform (Leica Biosystems, Nussloch GmbH) with bond polymer refine detection kit and bond polymer refine red detection kit (DS9800 and DS9390, respectively, Leica Biosystems). Briefly, 4-micron sections from tissue blocks were taken on coated slides. These were then deparaffinized, hydrated and blocked with hydrogen peroxide. Heat induced antigen retrieval was achieved using appropriate buffer for each antibody as per optimized protocol in control tissue. Slides were incubated with primary antibody followed by secondary antibody. Staining was completed with diaminobenzidine (DAB) chromogen and haematoxylin was used as a counterstain.

To assess the tumor vascular normalization index (VNI), double sequential staining for endothelial cells and pericytes was performed using CD31 and alpha-smooth muscle actin (α-SMA), respectively. This was followed by visualization with alkaline phosphatase-based red (DS9390) and peroxidase-based diaminobenzidine polymer detection systems (DS9800), respectively. VNI was calculated as the percentage of CD31-positive cells which co-express α-SMA in relation to the total number of blood vessels in the entire biopsy. This index was used as an indicator of tumor vessel maturation [13]. D2-40 antibody was selected for marking the lymphatic vessels and analyzing the lymphatic vessel density (average number of vessels positive for D2-40 in the entire biopsy). To study the pharmacodynamic effect of sunitinib and bevacizumab on tumor cells, the expression of activated VEGFR2 (phosphorylated VEGFR2 at tyrosine phosphorylation sites 951 and 996 [Y951 and Y996]) on tumor cells was examined. VEGFR2 expression was semi-quantified by H score, which is the percentage of tumor cells staining positive multiplied by an intensity score (0: no staining, 1: weak staining, 2: moderate staining, 3: strong staining). The final score ranged from 0 to 300 [14]. Ki67 proliferation index of tumor was calculated as percentage of positively stained cells among the total number of malignant cells [15]. Details on the antibody clone, commercial supplier, dilution and antigen retrieval are provided in Table 1.

**Table 1:** Details of antibodies used in immunohistochemistry studies

Antibody	Clone	Manufacturer	Dilution	Antigen retrieval
CD31	JC70A	Dako	1:100	Citrate buffer, pH 6, 20 min
α-SMA	1A4	Dako	1:500	Citrate buffer, pH 6, 20 min
D2-40/podoplanin	D2-40	Dako	1:100	Citrate buffer, pH 6, 20 min
p-VEGFR2 951	Rabbit polyclonal	Invitrogen	1:50	Citrate buffer, pH 6, 20 min
p-VEGFR2 996	Rabbit polyclonal	Invitrogen	1:100	EDTA buffer, pH 9, 20 min
Ki67	MIB-1	Dako	1:100	EDTA buffer, pH 9, 20 min

### Evaluation of histological response on surgical specimens

After completing 4 cycles of ddAC chemotherapy, patients with non-metastatic cancer underwent lumpectomy or mastectomy and sentinel lymph node biopsy or axillary lymph node clearance. Scoring of histological response on the primary tumor was done using the 5-point scale Miller–Payne grading (MPG) classification which was based on comparing the tumor cellularity between baseline and post-C4 biopsy. Good histological response was defined as a score of ≥ 3, i.e., more than 30% reduction of tumor cellularity in post-treatment biopsies from baseline [16].

### Statistical analysis

All statistical analyses were performed using SPSS software version 20.0 (IBM, Armonk, NY, USA). Categorical variables were presented as frequencies and percentages, while continuous variables as mean with standard deviation (SD). Comparison of continuous data between groups was done using the non-parametric Wilcoxon signed rank test. Categorical data were compared using Chi-square test. Independent *t*-test was run for comparison of means. A p value of less than 0.05 was considered statistically significant.

## Results

A total of sixty-two subjects were recruited; 38 patients were enrolled into the Sunitinib Cohort and 24 into the Bevacizumab Cohort. The baseline demographic and tumor characteristics of the patients are summarized in Table 2. Median age of the entire cohort was 51 years (range 29–70). Majority of the patients were Chinese, had invasive ductal carcinoma, hormone receptor-positive cancer, and non-metastatic disease. There were no significant differences in demographic or baseline tumor characteristics between the Sunitinib and Bevacizumab Cohorts.

**Table 2:** Baseline clinical and pathological characteristics of patients in the two treatment groups

Characteristics/Variables	Number (percentage)		
	Sunitinib (*n* = 38)	Bevacizumab (*n* = 24)	*p* value
Age (years)			
Median	53.5	49.5	0.406
Range	30–69	29–70	
Race			
Chinese	24 (63.1)	16 (66.7)	0.679
Malay	5 (13.2)	5 (20.8)	
Indian	3 (7.9)	1 (4.2)	
Others	6 (15.8)	2 (8.3)	
Histological type of tumor			
Ductal	33 (86.8)	20 (83.3)	0.887
Lobular	3 (7.9)	2 (8.3)	
Others	2 (5.3)	2 (8.3)	
Histological grade of tumor			
Grade 1 (Well differentiated)	2 (5.3)	0	0.183
Grade 2 (Moderately differentiated)	10 (26.3)	11 (45.8)	
Grade 3 (poorly differentiated)	26 (68.4)	13 (54.2)	
Hormone receptor status			
ER and/or PR-Positive	29 (76.3)	18 (75)	0.906
ER/PR-Negative	9 (23.7)	6 (25)	
Clinical T stage of primary tumor			
T1	1 (2.6)	1 (2.6)	0.988
T2	21 (55.3)	13 (34.2)	
T3	10 (26.3)	6 (15.8)	
T4	6 (15.8)	4 (10.5)	
Clinical node status			
N0	10 (26.3)	10 (41.6)	0.569
N1	22 (57.9)	12 (50)	
N2	2 (5.3)	1 (4.2)	
N3	4 (10.5)	1 (4.2)	
Metastasis			
Present	5 (13.2)	2 (8.3)	0.559
Absent	33 (86.8)	22 (91.7)	

*ER* estrogen receptor, *PR* progesterone receptor

### Immunohistochemistry studies

**Fig. 1:**
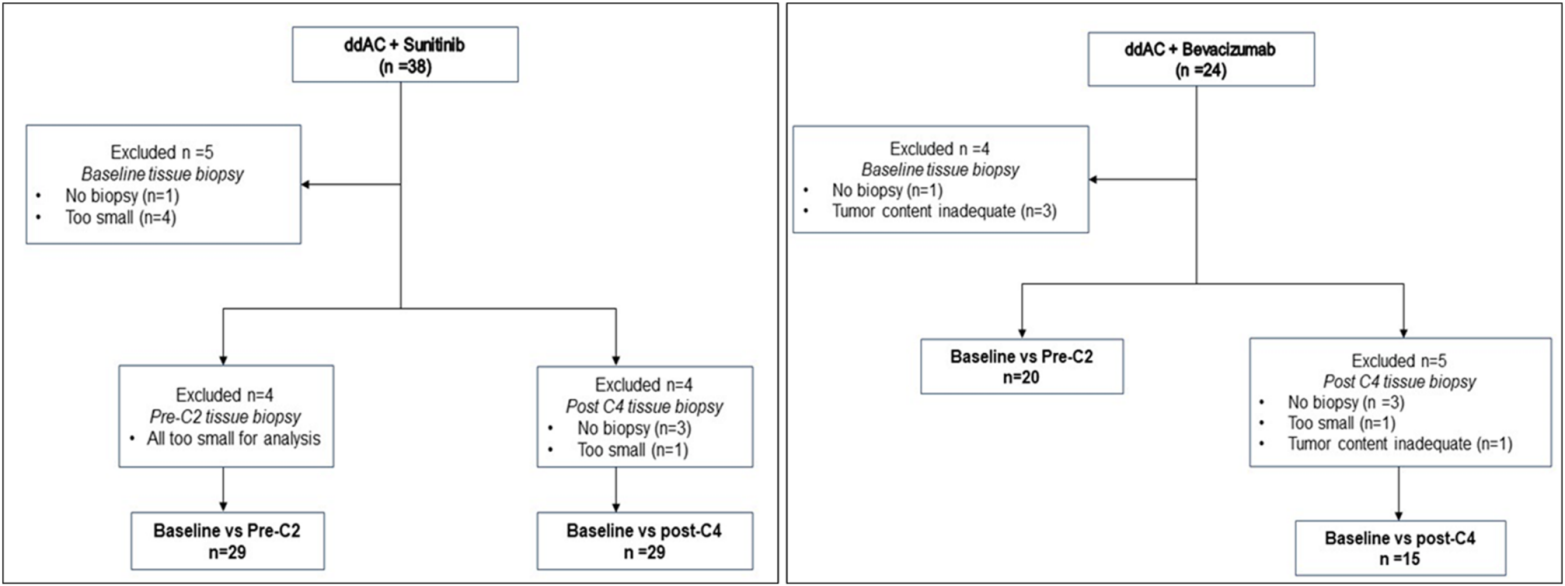
Consort diagram showing the selection of cases for immunohistochemical analysis. No biopsy—Surgical biopsy not taken at that timepoint, Too small—Tissue was very small for multiple sections required for histological and immunohistochemistry analysis, Inadequate—Biopsies which showed tumor content ≤ 10%

Out of the total 62 patients, a full set of pre-treatment, post-C1 and post-C4 tumor samples were available for IHC analysis for 29 of the 38 patients enrolled into the sunitinib cohort, and 15 of the 24 patients enrolled into the Bevacizumab cohort. An additional 5 patients enrolled into the bevacizumab cohort had pre-treatment and post-C1 samples without a post-C4 tumor sample. Reasons for incomplete tumor specimens for IHC analysis include no biopsy because of complete clinical response, core biopsy tumor specimen too small, or insufficient tumor content for IHC analysis (Fig. 1).

### Changes in lymphatic vessel density, vascular normalization index, Ki67 and p-VEGFR2 induced by Sunitinib versus Bevacizumab

**Fig. 2:**
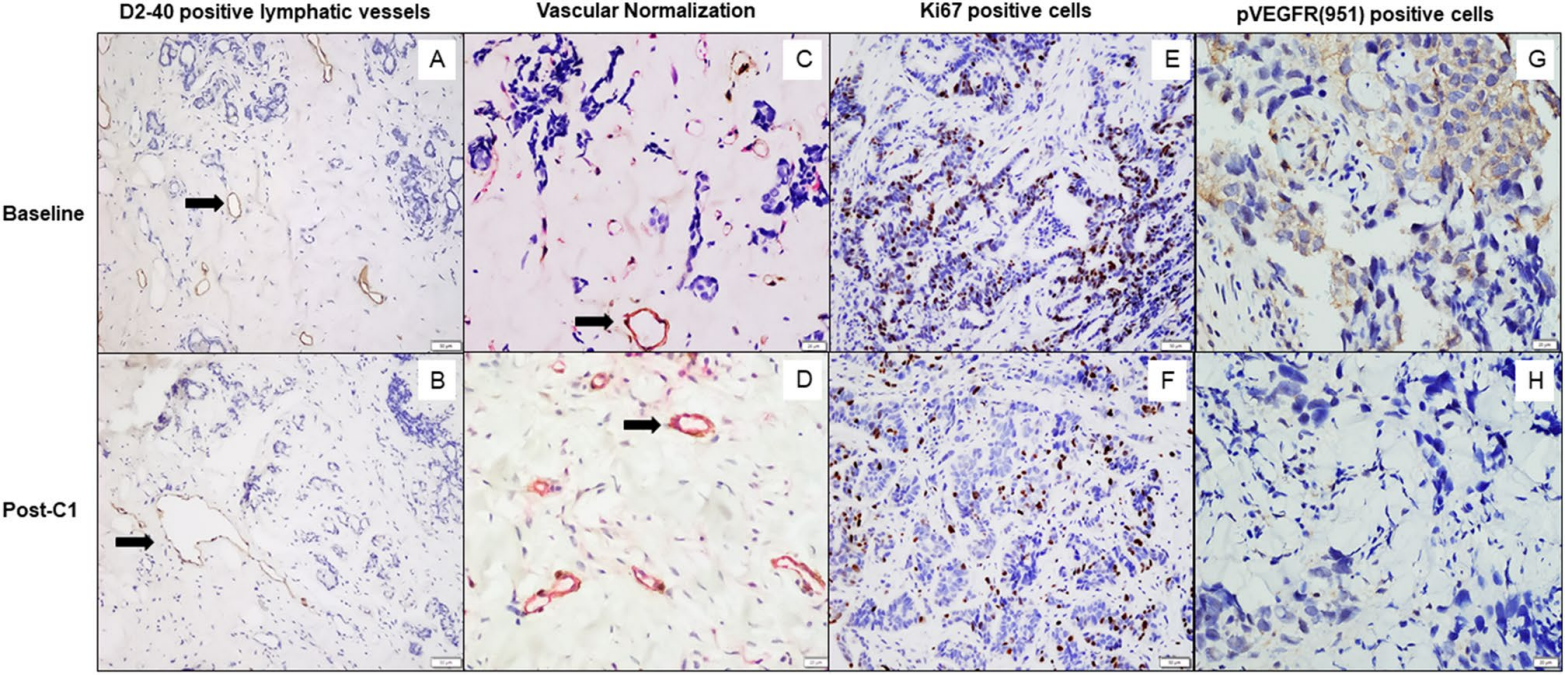
In a representative biopsy from a patient pre-treated with sunitinib, immunohistochemistry staining showing decrease in the number of D2-40-positive lymphatic vessels post-cycle 1 compared to baseline (Image **a** and **b**; × 200 magnification). Increase in vascular normalization index seen in post-cycle 1 where CD31 + vessels (in red) are covered with a layer of SMA + pericytes (in brown), in comparison to baseline (Image **c** and **d**; × 400X magnification). Reduction of Ki67 proliferation index in tumor cells post-cycle 1 compared to baseline (Image **e** and **f**; × 200 magnification). Decrease in expression of p-VEGFR2 (Y951) in tumor cells post-cycle 1 in comparison to baseline observed in a representative biopsy from patient pre-treated with bevacizumab (Image **g** and **h**; × 400 magnification)

**Fig. 3:**
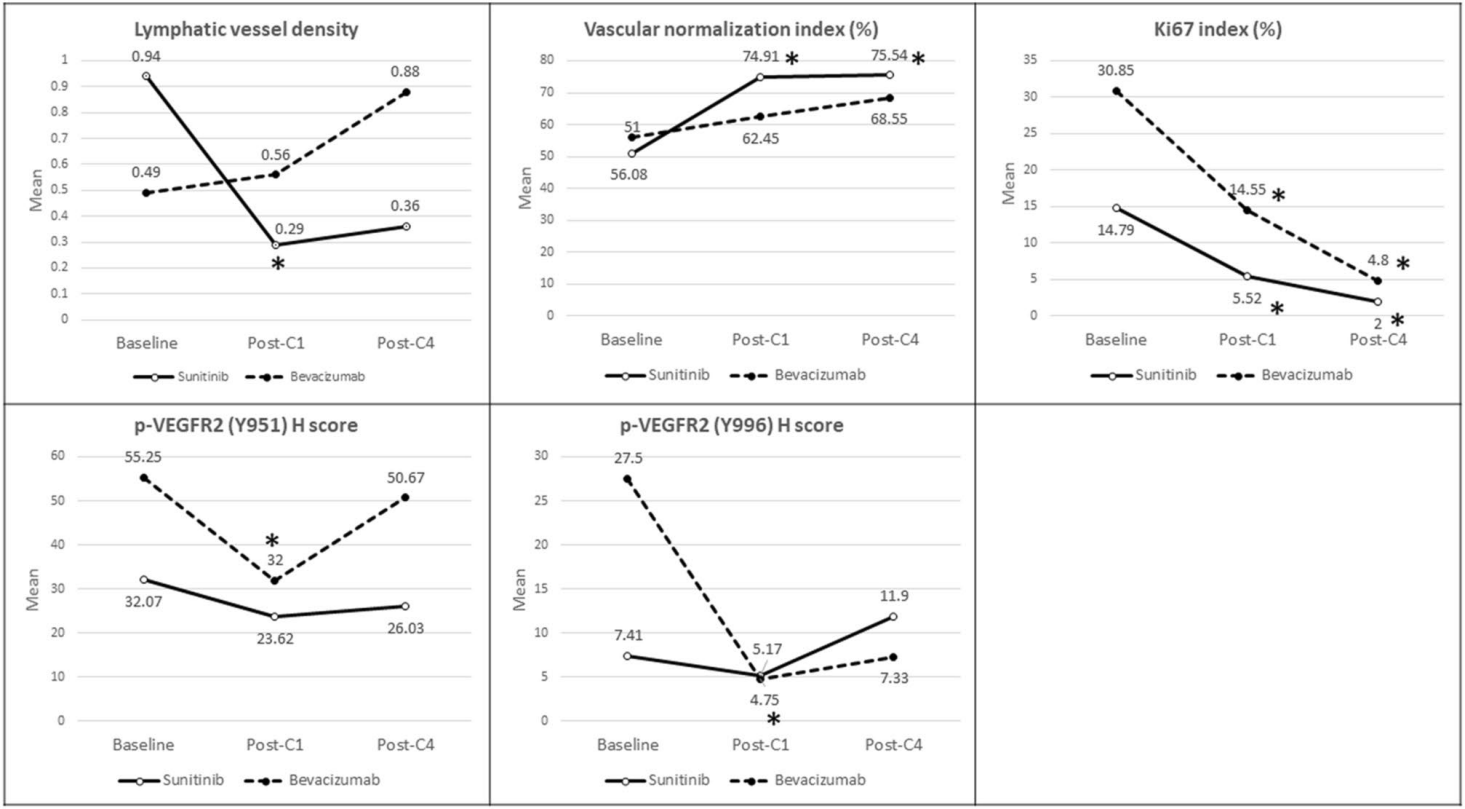
Graphical representation comparing the change in IHC parameters at cycle 1 and cycle 4 from baseline, between the two cohorts. In contrast to Bevacizumab, Sunitinib cohort showed a significant decrease from baseline in lymphatic vessel density after cycle 1 along with a sustained significant increase in vascular normalization index after cycle 1 and cycle 4. Bevacizumab cohort revealed a significant decrease in p-VEGFR2 (Y951 and Y996) expression in tumor cells post-cycle 1 from baseline, indicating its inhibitory effect on tumor growth. Both cohorts display significant decline in Ki67 tumor proliferation index after cycle 1 and 4; however, the reduction appears earlier and more marked in the sunitinib cohort. *Significant change from baseline (*p* < 0.05)

In the Sunitinib Cohort, there was a significant decrease in tumor lymphatic vessel density (LVD) after cycle 1 that persisted after cycle 4 chemotherapy compared to baseline (mean LVD 0.94 ± 1.39, 0.29 ± 0.45, 0.36 ± 0.58 for baseline, post-C1 and post-C4; *p* = 0.017 for baseline vs post-C1, *p* = 0.112 for baseline vs post-C4) (Fig. 2a, b). In contrast, there was a numerical increase in LVD that was not statistically significant after cycle 1 or cycle 4 chemotherapy compared to baseline observed in the Bevacizumab Cohort (Table & Fig. 3). While there were no differences in LVD at baseline among sunitinib-treated patients by clinical or pathological nodal status, there was a trend that those who were pathologically node-negative after neoadjuvant chemotherapy had lower LVD post-cycle 1 treatment than those who were pathologically node-positive (0.13 ± 0.17 vs 0.41 ± 0.54, *p* = 0.06).

Significant increase in Vascular normalization Index (VNI) was observed in the Sunitinib Cohort after one cycle of chemotherapy and that persisted after four cycles of chemotherapy, compared to baseline (mean VNI 51.00 ± 21.97%, 74.91 ± 18.93%, 75.54 ± 21.23% for baseline, post-C1 and post-C4; *p* < 0.001 for baseline vs post-C1, *p* = 0.001 for baseline vs post-C4) (Fig. 2c, d). While a similar trend was observed in the Bevacizumab Cohort, the differences were not statistically significant (Table 3, Fig. 3).

**Table 3:** Comparison of IHC parameters between the two treatment groups

IHC parameters	Sunitinib					Bevacizumab				
	Mean ± SD		*p* value^a^	Mean ± SD	*p* value^b^	Mean ± SD		p value^a^	Mean ± SD	*p* value^b^
	Baseline (*n* = 29)	Post-C1 (*n* = 29)		Post-C4 (*n* = 29)		Baseline (*n* = 20)	Post-C1 (*n* = 20)		Post-C4 (*n* = 15)	
LVD	0.94 ± 1.39	0.29 ± 0.45	**0.017**	0.36 ± 0.58	0.112	0.49 ± 0.72	0.56 ± 1.02	0.874	0.88 ± 1.40	0.528
VNI (%)	51.00 ± 21.97	74.91 ± 18.93	** < 0.001**	75.54 ± 21.23	**0.001**	56.08 ± 20.54	62.45 ± 26.71	0.070	68.55 ± 25.97	0.112
Ki67 index (%)	14.79 ± 23.81	5.52 ± 10.39	**0.027**	2.00 ± 7.54	**0.006**	30.85 ± 33.16	14.55 ± 22.96	**0.005**	4.80 ± 8.26	**0.021**
p-VEGFR2 (Y951) H score	32.07 ± 40.30	23.62 ± 34.25	0.245	26.03 ± 38.54	0.651	55.25 ± 41.15	32.00 ± 38.60	**0.008**	50.67 ± 41.82	0.819
p-VEGFR2 (Y996) H score	7.41 ± 10.05	5.17 ± 10.81	0.351	11.90 ± 22.33	0.500	27.50 ± 38.54	4.75 ± 9.79	**0.004**	7.33 ± 10.83	0.124

Bold indicates statistically significant values (*p* < 0.05)

^a^*p* value comparing the difference in means at post-C1 vs baseline

^b^*p* value comparing the difference in means at post-C4 vs baseline

In both the Sunitinib and Bevacizumab Cohorts, there was a significant decline in Ki67 proliferation index after cycle 1 and cycle 4 chemotherapy, although the decline appears quicker and more marked in the Sunitinib Cohort (mean Ki67 in Sunitinib Cohort 14.79 ± 23.81%, 5.52 ± 10.39, 2.00 ± 7.54% for baseline, post-C1 and post-C4; *p* = 0.027 for baseline vs post-C1, *p* = 0.006 for baseline vs post-C4; mean Ki67 in Bevacizumab Cohort 30.85 ± 33.16, 14.55 ± 22.96, 4.80 ± 8.26 for baseline, post-C1 and post-C4; *p* = 0.005 for baseline vs post-C1, *p* = 0.021 for baseline vs post-C4) (Fig. 2e, f) (Table 3, Fig. 3).

Expression of p-VEGFR2 on tumor cells was significantly reduced after cycle one chemotherapy in the Bevacizumab Cohort for both Y951 and Y996, although there appears to be a rebound in p-VEGFR2 after cycle 4 chemotherapy (mean *H* score [Y951] 55.25 ± 41.15, 32.00 ± 38.60, 50.67 ± 41.82 for baseline, post-C1 and post-C4; *p* = 0.008 for baseline vs post-C1, *p* = 0.819 for baseline vs post-C4; mean H score [Y996] 27.50 ± 38.54, 4.75 ± 9.79, 7.33 ± 10.83 for baseline, post-C1 and post-C4; *p* = 0.004 for baseline vs post-C1, *p* = 0.124 for baseline vs post-C4) (Fig. 2g, h). A similar trend was noted in the Sunitinib Cohort but the difference was not statistically significant (Table 3, Fig. 3).

### Clinical and pathological outcomes and correlation with IHC parameters

After 4 cycles of ddAC chemotherapy, 2.7% and 8.3% of patients in the Sunitinib and Bevacizumab Cohorts respectively achieved complete clinical response; 73% and 75% respectively achieved clinical partial response, while 24.3% and 16.7% achieved stable disease. No patient in either Cohort had clinical progressive disease. Patients with a complete or partial response were considered as good clinical responders, while those with only stable disease as poor clinical responders. Thirty-three patients in the Sunitinib Cohort and 17 patients in the Bevacizumab Cohort underwent surgery. No patient achieved pathological complete response. Using the MPG system classification, 66.7% and 76.5% in the Sunitinib and Bevacizumab Cohorts respectively achieved good histological response after 4 cycles of ddAC. No significant differences in clinical or histological responses were observed between the Sunitinib and Bevacizumab Cohorts (percentage good clinical responders in Sunitinib vs Bevacizumab Cohorts 75.7% vs 83.3%, *p* = 0.57; percentage good histological responders in Sunitinib vs Bevacizumab Cohorts 66.7% vs 76.5%, *p* = 0.47). There was a statistically significant difference in the post-C4 Ki67 index between clinical good vs poor responders in both cohorts (Sunitinib cohort, mean Ki67 index 0.74 ± 2.07% vs 8.20 ± 17.78% for good vs poor responders; *p* = 0.04; Bevacizumab cohort, mean Ki67 index 3.15 ± 4.75% vs 15.50 ± 20.50% for good vs poor responders; *p* = 0.044). However, there was no significant difference in the other IHC parameters (VNI, LVD, p-VEGFR2 H score) between good and poor clinical and histological responders in both the cohorts (Tables 4 and 5).

**Table 4:** Correlation of IHC parameters with good vs poor clinical response in the two treatment groups

IHC parameters at timepoints	Sunitinib (*n* = 28) ^a^			Bevacizumab (*n* = 20) ^b^		
	Mean ± SD			Mean ± SD		
	Good responder (*n* = 23)	Poor responder (*n* = 5)	*p* value	Good responder (*n* = 16) ^b^	Poor responder (*n* = 4) ^b^	*p* value
Baseline						
LVD	0.83 ± 1.29	0.80 ± 0.98	0.97	0.42 ± 0.51	0.75 ± 1.36	0.43
VNI (%)	52.88 ± 21.57	40.58 ± 25.37	0.27	53.93 ± 19.72	64.65 ± 24.59	0.36
Ki67 index (%)	14.00 ± 23.63	21.20 ± 28.19	0.55	27.31 ± 33.02	44.00 ± 38.73	0.35
p-VEGFR2 (Y951) H score	33.04 ± 42.04	26.00 ± 39.74	0.73	53.13 ± 41.42	63.75 ± 44.97	0.65
p-VEGFR2 (Y996) H score	8.04 ± 10.84	6.00 ± 6.51	0.69	29.38 ± 48.53	20.00 ± 16.33	0.67
Post-C1						
LVD	0.34 ± 0.48	0.12 ± 0.16	0.32	0.68 ± 1.11	0.05 ± 0.010	0.27
VNI (%)	74.61 ± 20.18	71.28 ± 9.75	0.72	66.92 ± 23.60	44.57 ± 34.65	0.13
Ki67 index (%)	4.70 ± 10.13	10.40 ± 12.28	0.28	10.61 ± 19.71	30.00 ± 31.62	0.13
p-VEGFR2 (Y951) H score	27.61 ± 36.30	10.00 ± 22.30	0.31	25.00 ± 34.83	60.00 ± 45.46	0.1
p-VEGFR2 (Y996) H score	5.65 ± 11.90	4.00 ± 5.47	0.76	5.94 ± 10.68	0	0.29
Post-C4^b^ (*n* = 15 for bevacizumab)						
LVD	0.44 ± 0.61	0.08 ± 0.09	0.21	0.64 ± 0.94	2.4 ± 3.39	0.1
VNI (%)	78.84 ± 16.06	75.49 ± 15.71	0.67	70.63 ± 23.92	55.06 ± 46.44	0.45
Ki67 index (%)	0.74 ± 2.07	8.20 ± 17.78	**0.04**	3.15 ± 4.75	15.00 ± 20.50	**0.04**
p-VEGFR2 (Y951) H score	27.17 ± 41.22	26.00 ± 29.66	0.95	43.08 ± 39.45	100.00 ± 14.14	0.71
p-VEGFR2 (Y996) H score	10.43 ± 19.93	21.00 ± 33.98	0.35	6.92 ± 10.90	10.00 ± 14.14	0.72

Bold indicates statistically significant values (*p* < 0.05)

^a^Clinical response was not available for one patient of the total 29 patients whose biopsy samples were analyzed at all time points

^b^In bevacizumab cohort, post-C4 biopsy IHC analysis was possible for 15 of total 20 patients. Of those 15 patients, 13 were good responders and 2 were poor responders

**Table 5:** Correlation of IHC parameters with good vs poor histological response in the two treatment groups

IHC parameters at time points	Sunitinib (*n* = 26)^a^			Bevacizumab (*n* = 16)^b^		
	Mean ± SD			Mean ± SD		
	Good responder (*n* = 17)	Poor responder (*n* = 9)	*p* value	Good responder (*n* = 12)^b^	Poor responder (*n* = 4)^b^	*p* value
Baseline						
LVD	0.88 ± 1.49	0.68 ± 0.83	0.72	0.56 ± 0.86	0.20 ± 0.28	0.42
VNI	56.18 ± 19.49	44.25 ± 21.42	0.16	60.24 ± 22.91	48.70 ± 10.29	0.35
Ki67 index	13.67 ± 21.64	21.11 ± 30.49	0.47	31.75 ± 30.06	24.00 ± 44.05	0.69
p-VEGFR2 (Y951) H score	36.47 ± 44.99	28.89 ± 37.23	0.67	57.08 ± 43.82	47.5 ± 40.30	0.7
p-VEGFR2 (Y996) H score	8.24 ± 9.00	7.78 ± 13.28	0.91	34.17 ± 46.16	12.5 ± 8.66	0.37
Post-C1						
LVD	0.41 ± 0.54	0.13 ± 0.26	0.16	0.85 ± 1.25	0.10 ± 0.20	0.26
VNI	75.34 ± 14.94	68.91 ± 24.67	0.41	55.18 ± 31.88	74.63 ± 5.07	0.25
Ki67 index	5.24 ± 9.82	7.89 ± 12.94	0.56	9.00 ± 13.06	27.5 ± 34.03	0.12
p-VEGFR2 (Y951) H score	26.18 ± 36.72	25.56 ± 35.04	0.96	31.67 ± 37.61	25.00 ± 43.58	0.77
p-VEGFR2 (Y996) H score	7.65 ± 13.36	2.22 ± 4.41	0.25	4..17 ± 9.96	7.50 ± 15.50	0.61
Post-C4^b^ (*n* = 11 for bevacizumab)						
LVD	0.37 ± 0.64	0.40 ± 0.55	0.92	0.92 ± 1.59	0.20 ± 0.34	0.47
VNI	80.66 ± 12.64	74.14 ± 22.05	0.34	71.13 ± 29.80	57.07 ± 30.51	0.5
Ki67 index	2.71 ± 9.62	1.33 ± 3.27	0.68	7.5 ± 10.35	0.67 ± 0.57	0.29
p-VEGFR2 (Y951) H score	32.67 ± 43.70	22.22 ± 31.92	0.53	43.75 ± 48.67	56.67 ± 41.63	0.69
p-VEGFR2 (Y996) H score	8.53 ± 15.48	22.22 ± 32.70	0.15	7.50 ± 11.67	6.67 ± 11.54	0.91

^a^Histological response could be analyzed in 26 of 29 patients in sunitinib cohort and 16 of 20 patients in bevacizumab cohort due to very little tissue remaining in block after prior sectioning for IHC analysis

^b^In bevacizumab cohort, post-C4 biopsy IHC analysis was possible for 11 of total 16 patients. Of those 11 patients, 8 were good responders and 3 were poor responders

## Discussion

Ever since the proposal of the “vascular normalization” theory, many preclinical observations show that the addition of anti-angiogenic agents to systemic chemotherapy leads to improved and uniform drug delivery and tumor control. The concept of presence of “normalization window” has provided insight into the probable benefit of administering low-dose and short-course anti-angiogenic drugs with chemotherapy instead of high and prolonged dosing [17, 18]. However, limited clinical data is available to prove the same. In this study we performed an immunohistochemistry analysis to investigate and compare the effects of two anti-angiogenic drugs, sunitinib and bevacizumab, that are commonly used in clinical practice. Doses of sunitinib was administered at a third of FDA approved dosing, and bevacizumab dosing was also lower than that studied in previous breast cancer trials including the RIBBON1 study to achieve a “vascular normalization” effect rather than anti-angiogenic effect [19]. We examined vascular normalization, lymphatic vessel density, tumor proliferation index and activated VEGFR2 status of tumor cells in both treatment groups.

Tumor vasculature normalization has been observed as early as 7 days after treatment [10]. In our previous study (Wong et al.) evaluating low-dose, short-course sunitinib prior to chemotherapy, serial tumor biopsies and DCE-MRI at baseline, 7 days post-treatment and 28 days post-treatment in a small cohort of patients had shown the normalization effects to occur at 7 days but sustained at 28 days post-treatment [11]. As it is logistically challenging to perform multiple serial tumor biopsies on a larger cohort of patients, in this current trial, we have chosen to take the second biopsy 2 weeks after cycle 1 chemotherapy + anti-angiogenic agent, which we believe is still early enough to observe normalization effects.

We demonstrated that in HER2-negative breast cancers, pre-treatment with low-dose short-course sunitinib leads to statistically significant increase in vascular normalization index in comparison to bevacizumab. Though both sunitinib and bevacizumab are capable of establishing a more mature vascular network in tumor microenvironment, sunitinib showed more promising results. Sunitinib led to almost 45% increase in VNI after one cycle of treatment compared to baseline although further increase from cycle 1 to cycle 4 was minor. On the other hand, pre-treatment with bevacizumab resulted in only ~ 10 to 20% increase in VNI post-cycle 1 and 4 that was not statistically significant. The lymphatic vasculature also plays an important role in tumor cell progression and metastasis. Invasion in lymphatic vessels has been found to be associated with increased risk of lymph node and distant metastasis thereby leading to poor survival in breast cancer patients [20]. In our study, sunitinib appeared to inhibit lymphangiogenesis leading to significant decline in LVD after 1 cycle of treatment. In contrast, bevacizumab pre-treatment actually led to a numerical increase in LVD, albeit not statistically significant.

We previously conducted a phase Ib/II trial in which subjects were randomized to chemotherapy with or without low-dose, intermittent sunitinib. IHC evaluation on serial tumor biopsies showed evidence of increased VNI and decrease in LVD after chemotherapy in patients randomized to receive sunitinib, but not in those treated with chemotherapy alone [11]. The observations in this current study in the Sunitinib Cohort are concordant with our previous findings [11]. Somewhat surprisingly, these results were not replicated in the Bevacizumab Cohort in our current study. In order to tilt drug effects toward more vascularization normalization than anti-angiogenic, we used a sunitinib dose that was one-third full dose and administered it for only 5–7 days prior to each 2-weekly cycle of chemotherapy instead of continuously. For bevacizumab, we used half dose (5 mg/kg every 2 weeks rather than 10 mg/kg) and administered it 1 week before chemotherapy rather than concurrently with chemotherapy. We postulate that the bevacizumab dose administered in our trial may still be too high, thus resulting in less prominent vascularization normalization effects than sunitinib.

The slight increase in LVD observed after bevacizumab treatment may be that bevacizumab largely sequesters VEGF-A, thereby blocking VEGF-A/VEGFR2 signaling. This may result in a compensatory increase in other VEGF ligands like VEGF-C by tumor cells which then bind to VEGFR-3 on lymphatic endothelial cells leading to lymphangiogenesis [21]. On the other hand, the prominent effects on VNI and LVD seen with sunitinib can be on account of its action on multiple tyrosine kinase receptors. Apart from VEGFR, inhibition of other signaling pathways like platelet-derived growth factor receptor (PDGFR), stem cell factor receptor (KIT), FMS-like tyrosine kinase 3 (FLT3), colony-stimulating factor 1 receptor (CSF-1R), and rearranged during transfection (RET) may have resulted in the supplementary effect on tumor proliferation, angiogenesis and lymphangiogenesis [22, 23].

Breast tumors are known to produce VEGF and also express VEGFR2 on their surface. This autocrine signaling is responsible for tumor cell growth and division. VEGFR2 signaling can be inhibited by directly blocking the receptor or by interfering with the binding to its ligand VEGF. This is an anti-angiogenic effect and can be affected by both sunitinib, which blocks tyrosine phosphorylation of VEGFR, and bevacizumab which binds to and neutralizes VEGF. In a study on mouse mammary tumor model, sunitinib-treated mice showed decreased levels of tumor p-VEGFR2 [24]. We similarly observed decreased expression of tumor cell VEGFR2 after one cycle of chemotherapy in patients pre-treated with sunitinib as well as bevacizumab proving that both drugs exert anti-angiogenic effects through inactivation of the VEGFR2 receptor on tumor cells. Intriguingly, p-VEGFR2 expression rebounded after 4 cycles of chemotherapy indicating that the action of anti-angiogenic agents in inhibiting the receptor activation on tumor cells could be of limited duration. Indeed, in an earlier phase Ib trial in breast cancer, we had observed sunitinib-induced normalization of tumor vasculature to occur as early as 24 h; yet in another phase II randomized trial, intermittent, low-dose sunitinib combined with up to 6 cycles of docetaxel did not improve response rates compared to docetaxel alone [11]. We hypothesize that while initial treatment with sunitinib does normalize tumor vasculature, repeated administration may conversely compromise normal tumor vasculature and eventually impair chemotherapy delivery. In fact, it may be possible that just a single cycle or two of sunitinib prior to starting chemotherapy may be sufficient to normalize tumor vasculature [25]. On the other hand, the group that received bevacizumab showed a significant decrease in VEGFR2 expression on tumor cells in comparison to sunitinib. It is possible that the dose of the drugs could affect this autocrine loop signaling of tumor cells; a lower than clinically approved dose of sunitinib was used in this trial, while the bevacizumab dose administered was within the clinically approved range, with the latter thus exerting greater anti-angiogenic than vasculature normalization effects. Also, it is possible that the primary action of sunitinib may have been on endothelial cells rather than tumor cells. Similar results have been reported in a study by Wedam et al., where bevacizumab was administered to locally advanced breast cancer patients (*n* = 21) and a significant inhibitory effect on tumor cell VEGFR2 expression (in both phosphorylation sites-Y951 and Y996) was demonstrated by IHC [26]. Collectively, these findings of decreased VEGFR2 expression together with lowered proliferation index suggests that both anti-angiogenic agents cause inactivation of VEGFR2 on tumor cells thus decreasing tumor cell proliferation although bevacizumab appears to exert a stronger effect than sunitinib on tumor cell VEGFR2 at the doses administered in this trial.

In the initial phase of study, both non-metastatic and metastatic patients were included, resulting in a small proportion (13% in sunitinib cohort and 8% in bevacizumab cohort) of the study population having metastatic disease. Our data and conclusions remained unchanged when the analysis was performed on only non-metastatic patients. While we believe that our study of local immunohistochemical changes of vascular effects of low-dose anti-angiogenic effects on the breast tissue should be largely unaffected by the systemic effects of chemotherapy and disease burden in other disease sites, this is nonetheless a potential limitation of this study. In addition, while there is no single perfect method of evaluating tumor vessel normalization, histopathological analysis is considered one of the standard methods to successfully demonstrate the morphology and density of mature and immature vessels, although difficulty in vascular function assessment and dynamic monitoring of the vascularization process within the tumor is a pitfall [13, 27].

## Conclusion

Immunohistochemistry analysis of serial tumor biopsies from patients with HER2-negative breast cancer who received lower dose sunitinib or bevacizumab before standard chemotherapy showed modulation of vessel morphology in tumor tissue along with suppression of tumor cell proliferation. Changes in LVD, VNI, Ki67 and p-VEGFR2, were generally early and observed after one cycle of treatment but tended to plateau with additional cycles of treatments. At the doses and schedule studied in this trial, sunitinib induced tumor vasculature normalization and inhibited lymphangiogenesis more prominently than bevacizumab, while bevacizumab demonstrated more significant effects on tumor VEGFR2 than sunitinib suggesting greater anti-angiogenic activity. Sunitinib with its more prominent vasculature normalization effects led to greater and sustained decline in tumor Ki67 than bevacizumab in this study, highlighting the promise of normalizing tumor vasculature to optimize chemotherapy delivery in breast cancer. The observation that vasculature normalization and anti-angiogenic effects plateaued or even rebounded with additional treatment cycles suggest that perhaps restricting the use of an anti-angiogenic agent to just the first one to two cycles of chemotherapy could be sufficient to exert the desired effects without the need to combine with all chemotherapy cycles. This strategy of more judicious combination of an anti-angiogenic agent with chemotherapy warrants further investigations.

## Data Availability

All data generated or analyzed during this current study are included in this published article.
